# TEX10 Promotes the Tumorigenesis and Radiotherapy Resistance of Urinary Bladder Carcinoma by Stabilizing XRCC6

**DOI:** 10.1155/2021/5975893

**Published:** 2021-12-20

**Authors:** Sheng Luo, Wenjin Wang, Jingfang Feng, Rui Li

**Affiliations:** ^1^Department of Urology, Xiangyang No. 1 People's Hospital, Hubei University of Medicine, Xiangyang, China; ^2^Department of Nephrology, Xiangyang No. 1 People's Hospital, Hubei University of Medicine, Xiangyang, China

## Abstract

Urinary bladder carcinoma refers to the commonest carcinoma with weak prognostic result for the patient as impacted by the limited treatment possibilities and challenging diagnosing process. Nevertheless, the molecular underpinning of bladder carcinoma malignant progression is still not clear. As a novel core part of pluripotency circuitry, testicular expression 10 (TEX10) plays an actively noticeable effect on reprogramming, early embryo development, and embryonic stem cell self-renewal. Nevertheless, TEX10 expressions and functions within bladder carcinoma are still not known. The present work is aimed at revealing TEX10 expression and biological function within urinary bladder carcinoma and elucidating the potential mechanisms. Results showed that TEX10 is abundant in urinary bladder carcinoma, and its protein level was related to poor disease-free survival in a positive manner. Reduced TEX10 level inhibited urinary bladder carcinoma cell proliferating process and metastasis in vitro and xenograft tumorigenicity in vivo. Notably, TEX10 might regulate carcinoma cell proliferating process and metastasis via XRCC6, thereby controlling the signaling of Wnt/*β*-catenin and DNA repair channel. Moreover, TEX10 gene knockout reduced the radiotherapy resistance of urinary bladder carcinoma. In brief, this work revealed that TEX10 could exert a significant carcinogenic effect on urinary bladder carcinoma tumorigenesis and radiotherapy resistance through the activation of XRCC6-related channels. Accordingly, targeting TEX10 is likely to offer a novel and feasible therapeutically related strategy for inhibiting urinary bladder carcinoma tumorigenicity.

## 1. Introduction

Bladder carcinoma ranked the tenth most frequent carcinoma worldwide has clinical manifestations and heterogeneous natural history [1]. Urothelial bladder carcinoma, featuring great recurrence rate, progression, and primary and acquired resistance to platinum-based therapy [2, 3], refers to the main histological subtype of bladder carcinoma, which brings considerable economic burden to the health care system, and has a significant impact on the quality of life and overall prognosis of patients [1]. With the aging of the population, the incidence rate of bladder carcinoma is on the rise [4, 5]. Current treatments for bladder carcinoma include a combination of surgery, targeted therapy, radiotherapy, and immunotherapy [6, 7]. Bladder cancer is considered a chromatin disease due to the abnormally high mutation rate of chromatin proteins, pointing to the importance of studying epigenetic disorders and the regulation prospects of this carcinoma [8–13]. At the molecular level, bladder carcinoma can be divided into six subtypes: papillary, unstable, nonspecific, squamous, neuroendocrine, and interstitial rich [14]. These subtypes have different patient outcomes, cell phenotypes, molecular characteristics, and genetic changes [14]. However, differences in treatment outcomes among patients with bladder carcinoma remain important clinical challenge [15–17]. Further in-depth research of the molecular mechanism is likely to help develop feasible therapeutically related strategies for bladder carcinoma.

TEX10, pertaining to the five friends of methylated chtop (chromatin-associated protein) as well as rix complexes, is critical to cell cycle regulation, ribosome biogenesis, and transcriptional regulation [18, 19]. Most importantly, TEX10 is a new stemness factor, which interacts with Sox2, thus involving in the establishment and maintenance of pluripotency [20]. Tex10 has an enrichment under the place of superenhancers (SEs) based on a manner relying on Sox2, while coordinating DNA demethylation and histone acetylation under the place of SEs. Tex10 is critical to the pluripotency network. Its effect within SE activity epigenetic control to determine cell fate is elucidated [20]. It is generally accepted that many pluripotent genes contribute to the initiation of tumorigenesis, metastasis progression, and multidrug resistance [21, 22]. The core factor of pluripotency transcription (Oct4) pertaining to embryonic stem cell has a frequent expression within hepatocellular carcinoma, and the relevant expressions are related to the mentioned pertaining to putative carcinoma stem cell (CSC) marker as well as CSC properties [23]. Moreover, Nanog is also related to the formation of tumor-initiating stem cell [24, 25]. With the mentioned observations in mind, we proposed that the dysregulated expression of TEX10 was also closely related to the pathogenesis and development of carcinomas. However, few studies on the functions of TEX10 have been reported in carcinomas, especially in bladder carcinoma. Thus, this work investigated the expression and biological function of Tex10 in bladder carcinoma.

## 2. Results

### 2.1. Elevated mRNA and Protein Content Achieved by TEX10 in Urinary Bladder Carcinoma Tissue

**Figure 1:**
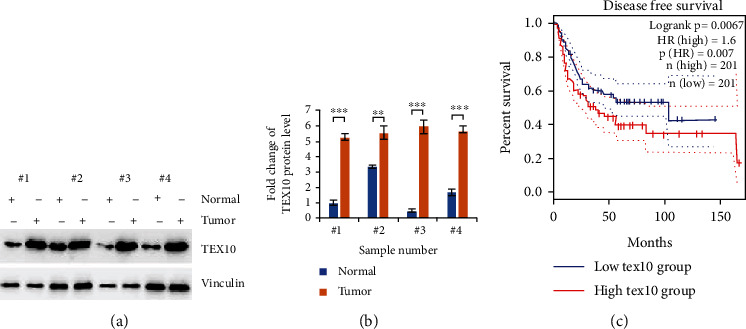
Identification of TEX10 related to DFS in urinary bladder carcinoma. (a, b) Four pairs of urinary bladder carcinoma tissue and nearby general tissues underwent immunoblotting investigation with the suggested antibodies. Shown is the mean ± SEM. ^∗^*P* < 0.05; ^∗∗^<0.01; ^∗∗∗^<0.001, Student's *t*-test. (c) Kaplan-Meier curves of DFS for 402 urinary bladder carcinoma patients of TCGA by adopting GEPIA online website under great or small TEX10 expressing levels.

The protein level of TEX10 was detected with immunoblotting in pairs of human normal tissues and urinary bladder carcinoma tissues, which were randomly chosen from four poorly differentiated urinary bladder carcinoma patients who underwent surgical resection in the Xiangyang No. 1 People's Hospital, China. In the normal tissue, the expressing state of TEX10 was low (Figures 1(a) and 1(b)). However, protein level high in cancerous tissue (Figures 1(a) and 1(b)), suggesting that protein level of TEX10 was upregulated in urinary bladder carcinoma. To further study the clinical significance of TEX10 level in patients with urinary bladder carcinoma samples, GEPIA online website (http://gepia2.carcinoma-pku.cn) was used to investigate relationship between expressing state of TEX10 and DFS for patients. As expected, the GEPIA online website investigation also suggested that the patients with a low-level TEX10 showed an improved DFS (Figure 1(c)).

### 2.2. TEX10 Promotes the Proliferating Process of Urinary Bladder Carcinoma Cell In Vitro

**Figure 2:**
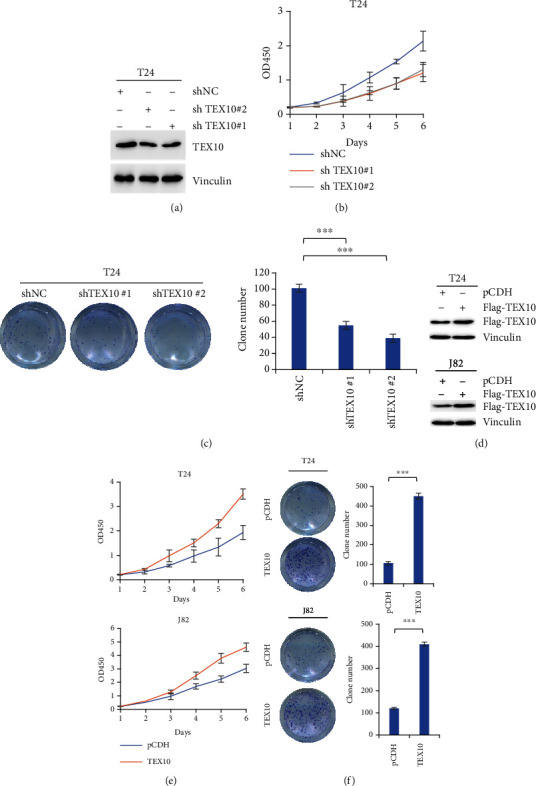
TEX10 promotes urinary bladder carcinoma cell proliferating process in vitro. (a) T24 cells with the steady expression of shNC, shTEX10 #1, and shTEX10 #2 underwent analyzed based on immunoblot using the suggested antibodies. (b) T24 cells with the steady expression of shNC, shTEX10 #1, and shTEX10 #2 underwent cell proliferating tests by CCK8 test. Shown is the mean ± SEM. ^∗^*P* < 0.05; ^∗∗^<0.01; ^∗∗∗^<0.001, Student's *t*-test. (c) T24 cells with the steady expression of shNC, shTEX10 #1, and shTEX10 #2 underwent cell proliferating tests by clone growth test. Shown is the mean ± SEM. ^∗^*P* < 0.05; ^∗∗^<0.01; ^∗∗∗^<0.001, Student's *t*-test. (d) T24 and J28 cells with the steady expression of pCDH and Flag-TEX10 were analyzed based on immunoblot. (e) T24 and J28 cells with the steady expression of pCDH and Flag-TEX10 underwent cell proliferating tests by CCK8 test. Shown is the mean ± SEM. ^∗^*P* < 0.05; ^∗∗^<0.01; ^∗∗∗^<0.001, Student's *t*-test. (f) T24 and J28 cells with the steady expression of pCDH and Flag-TEX10 underwent cell proliferating tests by clone development test. Shown is the mean ± SEM. ^∗^*P* < 0.05; ^∗∗^<0.01; ^∗∗∗^<0.001, Student's *t*-test.

For investigating the influence exerted by TEX10 on the growth of urinary bladder carcinoma cells, this study sets two TEX10 shRNAs to reduce TEX10 in T24 cell. According to Figure 2(a), the protein level of TEX10 noticeably decreased within T24 cell when TEX10 knockdown was achieved. Moreover, the TEX10 knockdown remarkably suppressed the proliferating process of T24 cells (Figures 2(b) and 2(c)). Furthermore, we overexpressed TEX10 in T24 and J82 cell (Figure 2(d)), and considerable promotions of growth were identified within T24 and J82 cell, as well as the overexpressed TEX10 (Figures 2(e) and 2(f)). For this reason, the mentioned results suggested that TEX10 facilitates the proliferating process of urinary bladder carcinoma cell in vitro.

### 2.3. A Positive Correlation between TEX10 Level and Urinary Bladder Carcinoma Growth In Vivo

**Figure 3:**
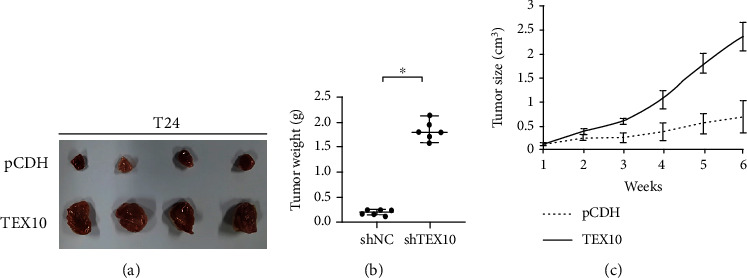
TEX10 accelerates urinary bladder carcinoma growth in vivo. T24 cells with the steady expression of pCDH and TEX10 received the injection to flank area of female BALB/c nude rats aged for 6 weeks (*n* = 6). After 6 weeks of injection, xenograft tumors received the harvesting. Images regarding harvested tumors (a), tumor weight (b), and tumor growth curves (c) are presented. Shown is the mean ± SEM. ^∗^*P* < 0.05; ^∗∗^<0.01; ^∗∗∗^<0.001, Student's *t*-test.

For more effectively studying the correlation of TEX10 and urinary bladder carcinoma growth, we used stable TEX10-overexpressing or control T24 cells into flank area of female BALB/c nude rats. After 6 weeks of injection, we harvested the tumor and reported that tumor sizes were noticeably larger within the TEX10-overexpressing rats than those in the controls (Figure 3(a)). Tumor weights from the TEX10-overexpressing group were obviously increased (Figure 3(b)), and the tumors in TEX10-overexpressing group displayed a higher growth rate (Figure 3(c)). Therefore, the mentioned result implied that TEX10 level was positively related to the urinary bladder carcinoma growth in vivo.

### 2.4. TEX10 Promotes the Migrating and Invading Processes of Urinary Bladder Carcinoma Cells

**Figure 4:**
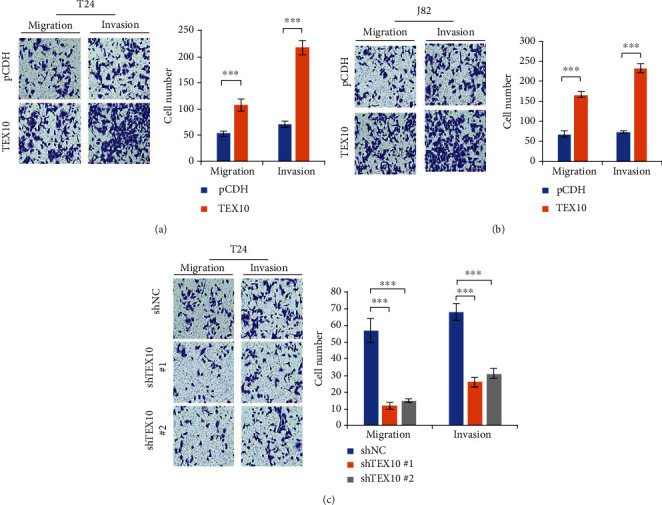
TEX10 promotes urinary bladder carcinoma cell migrating and invading processes. (a, b) T24 and J82 cells which stably expressed pCDH as well as Flag-TEX10 were conducted with Boyden's chamber migration tests (left) as well as Matrigel-coated invasion tests (right). Shown is the mean ± SEM. ^∗^*P* < 0.05; ^∗∗^<0.01; ^∗∗∗^<0.001, Student's *t*-test. (c) T24 cells which stably expressed shNC, shTEX10 #1, and shTEX10 #2 were conducted with Boyden's chamber migration tests (left) as well as Matrigel-coated invasion tests (right). Shown is the mean ± SEM. ^∗^*P* < 0.05; ^∗∗^<0.01; ^∗∗∗^<0.001, Student's *t*-test.

We investigated the role of TEX10 in the migrating and invading processes of urinary bladder carcinoma cells by Boyden's chamber tests and Matrigel-coated tests, respectively. We used the previously constructed TEX10-overexpressing and PCDH T24 stable cell line. In the study of the migrating and invading capabilities of TEX10-overexpressing T24 cells, the number of migrated cells increased almost twofold in contrast with controls, and the number of invasive cells noticeably rose (Figure 4(a)). Similar increases of the amounts of cells under migration and invasion were also found in TEX10-overexpressing J82 cells (Figure 4(b)), whereas both the amounts of cells under migration and invasion were found to dramatically decrease in T24 cells after TEX10 knockdown when compared with controls (Figure 4(c)). All the mentioned results suggested that the low level of TEX10 inhibited the migrating and invading behaviors of urinary bladder carcinoma cell.

### 2.5. TEX10 Advances the Tumorigenesis of Urinary Bladder Carcinoma by Increasing XRCC6 Expressing Level for Enhancing the Signaling of Wnt/*β*-Catenin

**Figure 5:**
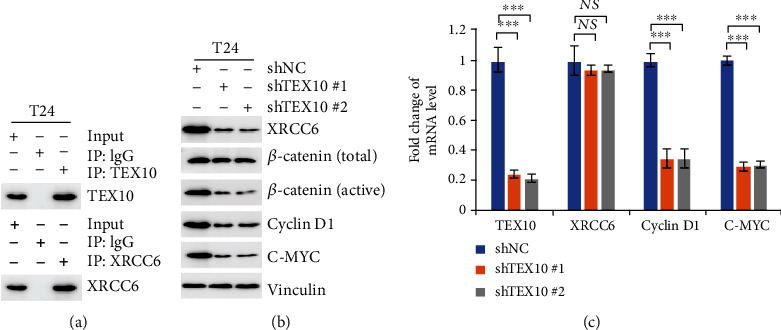
TEX10 regulates the level of XRCC6 and its downstream. (a) T24 cells were performed for immunoprecipitation with the TEX10 or XRCC6 antibodies, followed with immunoblot with suggested antibodies. (b) T24 cells with the steady expression of shNC, shTEX10 #1, and shTEX10 #2 underwent immunoblot using the suggested antibodies. (c) T24 cells with the steady expression of shNC, shTEX10 #1, and shTEX10 #2 underwent analyses by real-time PCR. Shown is the mean ± SEM. ^∗^*P* < 0.05; ^∗∗^<0.01; ^∗∗∗^<0.001, Student's *t*-test.

For more specifically characterizing the regulating influence exerted by TEX10 onto cell proliferating process, this study adopted a bioinformatics platform (BioGRID) under the integration to exploring the network of protein interaction (https://thebiogrid.org) for predicting TEX10 partner. XRCC6 might be a binding partner for TEX10. For achieving the mentioned aim, the authors tested of XRCC6 could interact with TEX10. Based on coimmunoprecipitation with an anti-XRCC6 and anti-TEX10 antibody, the interacting process of TEX10 and XRCC6 under the level of endogenous protein was verified within T24 cells. XRCC6 was reported to have tumor-promoting effect via Signaling of Wnt/*β*-catenin channel [26]. Then, we explored the potential mechanism of TEX10 by focusing on XRCC6 and Wnt/*β*-catenin channel in T24 cell. As TEX10 was silenced, XRCC6 protein content noticeably declined (Figure 5(b)). The mentioned finding was likely to suggest that TEX10 elevates the protein level of XRCC6. Moreover, according to the observation, active *β*-catenin protein content markedly declined in TEX10-silenced cell, while the overall *β*-catenin protein level kept stable in the mentioned cell, suggesting that the TEX10 knockdown downregulates *β*-catenin activation in urinary bladder carcinoma. Wnt/*β*-catenin channel critically impacts tumorigenesis promotion [27, 28]. Furthermore, we assessed the expressing states of cyclin D1 and c-Myc, acting as Wnt/*β*-catenin channel's downstream target. Figure 5(b) reveals that TEX10's, cyclin D1's, and c-Myc's mRNA states in the TEX10-silenced cells were dramatically decreased (Figure 5(c)), whereas the mRNA states of XRCC6 did not change in the mentioned cells, which suggested that TEX10 regulates level of XRCC6 in posttranscriptional level but not in mRNA state. Accordingly, the mentioned result gave the vital clue that TEX10 was likely to facilitate urinary bladder carcinoma tumorigenesis by increasing XRCC6 level to enhance the Signaling of Wnt/*β*-catenin.

### 2.6. TEX10 Promotes Efficiency of NHEJ through Regulating XRCC6

**Figure 6:**
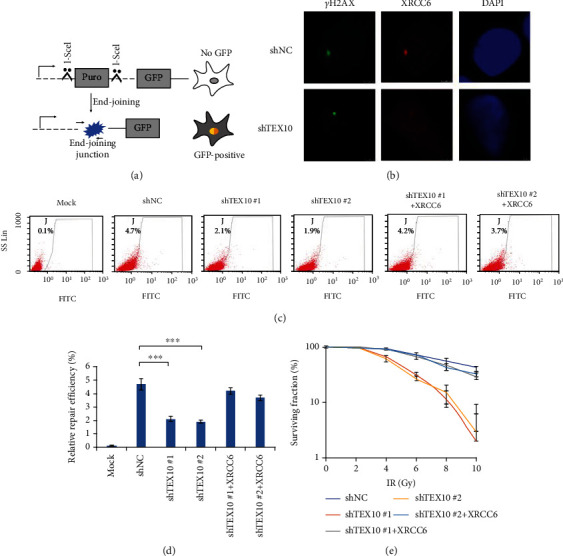
TEX10 promotes NHEJ efficiency. (a) Diagram of NHEJ. Scissors indicate the cut sites of I-Sce I. The cut sites were cleaved after I-Sce I expression. GFP was expressed after NHEJ repair completion. (b) Fluorescent observation of DSB from T24 cells as suggested after I-Sce I expression. (c, d) Efficiency of the NHEJ repairs analyzed with flow cytometry. The percentage of GFP expression was regarded as repair completion for NHEJ repair tests. Shown is the mean ± SEM. ^∗^*P* < 0.05; ^∗∗^<0.01; ^∗∗∗^<0.001, Student's *t*-test. (e) T24 cells received the treatment by using increasing doses of IR and subjected to survival tests by CCK8 test. Shown is the mean ± SEM. ^∗^*P* < 0.05; ^∗∗^<0.01; ^∗∗∗^<0.001, Student's *t*-test.

Given the impact of XRCC6 on NHEJ and DNA-end resection, we tended to determine whether TEX10 protein could influence the NHEJ processes. To explore this, we utilized a cell system with DSB in a defined genomic area able to be attributed to I-SceI endonuclease expression (Figure 6(a)). First, we used I-SceI to generate a DSB in T24 cells and examined *γ*H2AX localization to represent DSB site. As shown in Figure 6(b), XRCC6 localized to DSBs and colocalized with *γ*H2AX. Interestingly, TEX10 knockdown abolished DSB localization of XRCC6 (Figure 6(b)). The results showed that stabilization of XRCC6 by TEX10 is important for its recruitment to DSBs. When DSB repair is completed, cells will express GFP proteins according to I-SceI system. Therefore, NHEJ efficiency was assessed based on FACS investigation for GFP-positive cell. According to the expectation, Mock cells had rare GFP; this was able to be detected. As opposed to the mentioned, nearly 4.7% cell with the expression of I-SceI endonuclease was GFP positive. TEX10 knockdown by shRNAs noticeably decreased GFP-positive cell rate, and such an effect was partly remedied by reintroducing XRCC6 to cell under TEX10 knockdown (Figures 6(c) and 6(d)). The mRNA expressing state of TEX10 and XRCC6 is shown in Fig. S1A and Fig. S1B. Together, the mentioned data demonstrated that TEX10 promoted NHEJ repair of DSB via XRCC6. To evaluate whether NHEJ impairment following TEX10 depletion improves sensitive property toward IR, CCK8 tests were made with T24 cells and J82 cells with the steady expression of shNC and shTEX10 in XRCC6 reoverexpression presence or absence to evaluate sensitivity of cells to IR. As revealed from the result, TEX10 knockdown increased the sensitive property exhibited by cells to IR but rescued by reoverexpression of XRCC6 (Figure 6(e) and Supplementary Fig. S1C).

## 3. Discussion

Urinary bladder carcinoma, occupying the most portion of bladder carcinoma cases [8], lacks of effective diagnosing and treating strategies. Thus, useful means is urgently required for improving the diagnosis and therapy of urinary bladder carcinoma, which is noticeably based on the molecular mechanism clarification of urinary bladder carcinoma.

TEX10 has close relationships with the occurrence, development, and drug resistance of multiple carcinomas. Previous studies have reported that TEX10 participates in several regulation processes, including controlling ribosome biogenesis, which is strongly linked with carcinoma development [29, 30], and regulating cell cycle, critically impacting malignant progression of carcinomas [31, 32]. However, its detailed mechanism in bladder carcinoma is still not clear. Herein, we deeply investigated the underlying mechanism of TEX10 in urinary bladder carcinoma for providing valuable supports to the researches on the urinary bladder carcinoma. TEX101, belonging to the same family with TEX10, was also documented in several tissues' carcinoma cell in HNSCC patients, while the general squamous epithelium had the immunonegative characteristic. TEX101 refers to a new carcinoma-correlated protein, with a probably promising application to be a marker in terms of HNSCC prognosis/diagnosis. However, overexpression of urothelial carcinoma-associated 1*α* (UCA1*α*), which promotes bladder carcinoma progress, decreased the level of TEX101 in bladder carcinoma. It indicates that TEX101 and TEX10 play different roles in bladder carcinoma.

First of all, we found that TEX10 protein level rose noticeably within the human urinary bladder carcinoma tissue in contrast with the normal tissue in two sets. The mRNA state of TEX10 was also upregulated in the urinary bladder carcinoma tissue as compared with that within the general counterpart. Moreover, the low TEX10 expressing state presented an improved DFS in urinary bladder carcinoma patients. After TEX10 knockdown in the bladder carcinoma cell lines, the expression of TEX10 was dramatically reduced, and the growth of the mentioned cells was suppressed. On the contrary, the overexpression of TEX10 promoted the proliferation of urinary bladder carcinoma cell. Furthermore, the overexpression of TEX10 was observed to notably contribute to the tumor growth in the rats with urinary bladder carcinoma. By performing Matrigel-coated and Boyden's chamber tests, we discovered that the low level of TEX10 inhibited the migrating and invading processes of urinary bladder carcinoma cell. Combining the above findings, TEX10 was critical to the tumorigenesis of urinary bladder carcinoma.

The level of Ku proteins has been reported to have a delicate balance, overexpression of Ku proteins promotes carcinogenic phenotypes, including excessive proliferation and antiapoptosis, while the low level of Ku proteins leads to genomic instability and tumorigenesis [33]. Ku proteins include XRCC6/Ku70 and XRCC5/Ku80 and XRCC6 and XRCC5 as two subunits form a heterodimeric protein [34]. XRCC6 critically impacts chromosomal integrity and cell survival regulation [35]. Such a versatile regulating protein participates into a variety of nuclear processes, such as DNA repair, telomere maintenance, and apoptosis [36–39]. The DNA double-strand break (DSB) repair channels are essential for the maintenance of eukaryote genetic integrity, including homologous recombination and nonhomologous end joining (NHEJ) [33, 40]. Ku proteins as the critical NHEJ factors participate in the DNA NHEJ DSB channel. Importantly, XRCC6 binds to DSB ends with XRCC5 to correct NHEJ, thus activating DNA-dependent protein kinase [34, 41]. Recent evidences have shown that XRCC6 has a close participation in tumor appearance and growth and has great potential as an anticarcinoma drug candidate [42]. Therefore, understanding the relationship between various functions of XRCC6 and carcinogenesis of bladder carcinoma may contribute to the development of new anticarcinoma agents against bladder carcinoma.

XRCC6 is stated to be closely related to the carcinogenesis [43]. Interestingly, we discovered the noticeable relation of TEX10 and XRCC6 in urinary bladder carcinoma that TEX10 promotes the level of XRCC6. Furthermore, this study confirmed that TEX10 silencing inhibited the *β*-catenin and level activation of cyclin D1 and c-Myc, demonstrating that TEX10 might promote the tumorigenesis of urinary bladder carcinoma via increasing XRCC6 level to enhance the signaling of Wnt/*β*-catenin. Although TEX10 positively regulated the XRCC6 level and signaling of Wnt/*β*-catenin in urinary bladder carcinoma according to this work, the underlying molecular mechanisms involving TEX10, XRCC6, and Wnt/*β*-catenin channel remain to be further identified.

XRCC6 is also known as an important factor in the NHEJ process, which is responsible for the integrity of eukaryote genetic. Importantly, our study verified that TEX10 had great impact on the NHEJ process by regulating XRCC6. The mentioned findings revealed that TEX10 promotes NHEJ repair of DSB through enhancing XRCC6 level. Moreover, we found that the TEX10 knockdown remarkably improved the sensitivity of urinary bladder carcinoma cells to IR. However, this phenomenon would be reversed by the reoverexpression of XRCC6. Therefore, TEX10 might be a promising radiosensitization drug target for urinary bladder carcinoma treatment via mediating NHEJ impairment via XRCC6.

It is well-known that Wnt/*β*-catenin channel is a notable system to promote CSCs tumorigenicity and self-renewal within various carcinomas [44–46]. Nevertheless, rare study investigated the correlations of TEX10 and Wnt/*β*-catenin channel within urinary bladder carcinoma. Thus, studying the association of TEX10 and Wnt/*β*-catenin channel is beneficial to comprehend the underlying mechanism of TEX10 in urinary bladder carcinoma.

In conclusion, our study manifested that TEX10 serves as a critical role in bladder carcinoma tumorigenesis. And TEX10 presented great possibility to be one new and vital therapeutically related target for urinary bladder carcinoma treatment.

## 4. Materials and Methods

### 4.1. Ethical Approval

The patient gave the informed consent in terms of tissue sample diagnosing and study before enrollment. This work received the approval from the Ethics Committee of Xiangyang First People's Hospital. All animal tests received the approval from the Animal Protection and Use Committee of Hubei Medical University. Next, we made broad attempt for minimizing the suffering of animals applied.

### 4.2. Cell Lines and Cell Culture

Cell Resource Center of the Shanghai Institute of Life Sciences, Chinese Academy of Sciences, provided the cell line of T24 and J82, which received the authentication through short-tandem repeat test. The respective cell line had no mycoplasma and received the culture within RPMI-1640 media (Gibco; Thermo Fisher Scientific, Inc.) covering 1% penicillin-streptomycin (Gibco; Thermo Fisher Scientific, Inc.) and 10% fetal bovine serum (Gibco; Thermo Fisher Scientific, Inc.) within an incubating device under the temperature of 37°C based on 5% CO_2_.

### 4.3. Construction of Lentivirus and Stable Transfection into Cell Lines

Genechem Co., Ltd. built the lentiviral vector that encoded TEX10 gene of human. shNC and shTEX10 express lentiviral vector coding shRNA that targets shRNA and TEX10 negative control, separately. The target sequence included shTEX10#1, 5′-AGCTACTGCCCTCCGAATTTA-3′; shTEX10#2 5′-GATAGAACACTTCCGACAAAC-3′ as well as shNC, 5′-TTCTCCGAACGTGTCACGT-3′. The T24 cells received the transfection by exploiting recombinant lentivirus-transducing unit under polybrene (6 *μ*g/ml) in accordance with producer's guideline, and steady TEX10 knockdown clone (shTEX10 #1, shTEX10 #2) was adopted by using 2.5 *μ*g/ml puromycin (A1113803; Gibco; Thermo Fisher Scientific, Inc.). The taken knockdown cell pool received the collection and culture in 10 passages to be experimentally processed later. Tables S1 and S2 elucidate information concerning expression construct and the primer applied in terms of molecular cloning.

### 4.4. Coimmunoprecipitation Test and Immunoblotting

In terms of immunoblot investigation, this study employed RIPA lysis buffer under the modification (1 mM EDTA, 150 mM NaCl, 0.25% sodium deoxycholate, 1% Nonidet P-40, pH = 7.4, and 50 mM Tris-HCl) as supported by phosphatase and protease inhibitor (Bimake, Houston, USA) for lysing cell. We employed the reagent of BCA protein test (Yeasen, Shanghai, China) for detecting protein concentration. Cellular extract received the first decomposition based on SDS-PAGE prior to the use in PVDF membrane (Millipore, Billerica, USA). Next, they received the incubation with the suitable primary antibody. Subsequently, we exploited improved chemiluminescent substrate tool (Yeasen) for analyzing the particular suggested antibody signal. In terms of immunization-based precipitation of endogenous protein, the researchers employed primary antibody or control IgG for the incubation of cell extract within the incubating device under the rotation throughout the night at 4°C. Afterward, the resultant received the 3 h incubating process by exploiting protein A/G magnetic bead (Sigma-Aldrich, St. Louis, MO, USA). The lysis buffer was used to wash the immunoprecipitates for three times before immunoblotting investigation.

### 4.5. EJ5-End Joining Test

T24 cell received the stable transfection by employing with pimEJ5GFP (AddGene 44026). In brief, pimEJ5GFP received the digestion by exploiting XhoI, as well as the transfection to T24 cell. Steady transfected cells received the selection and maintenance within media covering 2 *μ*g/ml puromycin. To observe DSB localization, stable shNC and shTEX10 cell with the EJ5 reporter received the transfection by using pCBASceI (AddGene 26477), the 12 h incubating process to allow for plasmid expressions and subsequently the observation of DSB localization. To measure NHEJ efficiency, stable shNC and shTEX10 cell with the EJ5 reporter received the transfection by using pCDH and XRCC6 expression plasmids, then the 24 h incubating process for enabling plasmid expressions. Cell received the transfection by using pCBASceI (AddGene 26477), expressing I-SceI, and the 24 h incubation. Next, cell received the harvesting process, the washing process by using PBS, and the resuspending process within PBS. GFP received the detection based on cell sorting under the activation from fluorescence (FACS Calibur, Becton-Dickinson).

### 4.6. Reverse Transcription Quantitative Polymerase Chain Reaction (RT-qPCR)

Overall RNA received the extraction from the tissue by exploiting TRIzol reagent (Invitrogen; Thermo Fisher Scientific, Inc.) and reverse transcription with the use of the ThermoScript RT-PCR mechanism (cat. no. 11731015; Invitrogen; Thermo Fisher Scientific, Inc.) in accordance with procedure's guideline. The researchers carried out qPCR under SYBR-Green (cat. no. 1708886; Bio-Rad Laboratories, Inc.) onto one iQ5 Multicolor Real-Time PCR Detection mechanism (Bio-Rad Laboratories, Inc.). PCR condition included at the temperature of 95°C for 5 min, accompanied with 40 cycles at the temperature of 95°C for 30 s, as well as at the temperature of 57°C for 30 s. GAPDH acted as an internal control group. Overall reactions were made three times independently and received the quantification by using 2 − ΔΔCq [47].

### 4.7. Western Blot Investigation

T24 cells with the steady expression of shTEX10 or shNC received the collection and lysing process by exploiting radioimmunoprecipitation test buffer (Keygen Biotech) as supported by protease inhibitor cocktail (Roche Applied Science). Protein of the lysed cell received the fractionation with the use of 10% SDS-PAGE and then the transfer to nitrocellulose membrane (Hybond C; GE Healthcare Life Sciences). Nonspecific binding sites received the blocking process by adopting 5% nonfat milk under the ambient temperature for 1 h, and the membrane received the incubation by exploiting the antibody below throughout the night at 4°C: vimentin (cat. no. M1412-1; dilution, 1 : 1,000) (all from HuaBio Inc.), cyclin D1 (cat. no. ET1601-31; dilution, 1 : 1,000), *β*-catenin (cat. no. ET1601-5; dilution, 1 : 1,000), XRCC6 (cat. no. ab83501; dilution, 1 : 1000; Abcam), and anti-TEX 10 (cat. no. 17372-1-AP; dilution, 1 : 1000; Proteintech Group, Inc.). Membrane received the washing and incubating process by exploiting goat anti-mouse (cat. no. A4416; dilution, 1 : 10,000) antibody or goat anti-rabbit under the conjugation of HRP (cat. no. A4914; dilution, 1 : 10,000) (Sigma-Aldrich; Merck KGaA). The band received the visualization with the use of improved chemiluminescence reagent (cat. no. WP20005; Thermo Fisher Scientific, Inc.).

### 4.8. Cell Proliferating Test

T24 and J82 cell proliferation received the measurement with the use of cell count methods. For cell counting kit-8 (CCK-8) test (Keygen Biotech), the cells (2 × 10^3^/well) received the seeding process to 96-well plate and the daily observation, and at particular time-point, 10 *μ*l CCK-8 and 100 *μ*l fresh medium received the addition into the respective well. After the incubating process for 1.5 h under the temperature of 37°C, with the use of a microplate reader (ST-360; KHB), the absorbance received the measurement under 450 nm. The respective condition was tested three times independently. In terms of colony-formation test, 1 × 10^3^ stable cell received the seeding process into six-well plate and then the 14–21-day culture. Next, the cell received the fixing process by employing 4% paraformaldehyde and the staining process by adopting crystal violet staining solution. Visualize colony received the counting process.

### 4.9. Flow Cytometric Evaluation

The cell received the collection and washing by exploiting with ice-cold PBS. Based on an Annexin V-FITC reagent tool (cat. no. A211-01; Vazyme Biotech Co., Ltd.), we carried out an investigation. The cells received the resuspending process into a final concentration of 1 × 10^6^ cells/ml in Annexin V-binding buffer and the 15 min incubation by employing AnnexinV-FITC under propidium iodide under the temperature of 4°C. Samples received the analysis with the use of a BD FACSCalibur flow cytometer (BD Biosciences), and by employing FlowJo software (FlowJo 10.5, LLC), the following investigation were carried out.

### 4.10. In Vitro Migrating and Invading Tests

The migrating or invading tests were carried out with the use of polycarbonate Transwell filter chambers (8 *μ*m pore size; cat. no. 3422; Corning Inc.) and the inserts received the coating by using or not using Matrigel® (BD Biosciences). The shNC- or TEX10 shRNA-transfected T24 or J82 cells (5 × 10^4^) within 100 *μ*l serum-free medium received the introduction into the top chamber, but 500 *μ*l medium with 10% serum was added into the bottom chamber. Following incubation for 48 h at 37°C, all of the nonmigrating or noninvading cells were removed, and cells on the lower membrane of the inserts were stained with 0.1% crystal violet (KGA229, Keygen Biotech) under the ambient temperature for 5 min. Under a microscope (ECLIPSE TS100, Nikon), the number of cells under the migration or invasion were counted, and images were taken. The respective experiment-related condition was performed with triplicate filters, and the experiments were repeated 3 times.

### 4.11. Radiation Sensitivity Test

The T24 or J82 cells (1 × 10^4^/well) were seeded into 96-well plates and a serial dose of IR ranging from 0 to 10 gy was treated to the shNC or shTEX10-transfected T24 or J82 cells after 24 h. Cell proliferating process was measured with a CCK-8 test (KGA317s; Keygen Biotech) as described above.

### 4.12. Statistical Investigation

The experimental procedures were carried out in no less than three single experimental procedures in an independent manner, and all data were expressed as the means ± standard deviation. The SPSS 20.0 software (IBM Corp.) was used for statistical investigation. A two-tailed unpaired Student's *t*-test with Welch correction or one-way ANOVA followed by the Bonferroni test (for multiple comparisons) or Dunnett's adjustment (for dose-response effects) was adopted for determining the statistical significance of the differences in the measured variables. A value of *P* < 0.05 was considered with statistical significance.

## Data Availability

The datasets generated and/or analyzed in this study are available from the respective author upon reasonable request.

## Abbreviations

shTEX10: shRNA targeting TEX10
shNC: shRNA negative control
NHEJ: Nonhomologous end joining
DSB: Double-strand break
CSC: Carcinoma stem cell
DFS: Disease-free survival
RT-qPCR: Reverse transcription quantitative polymerase chain reaction.

## Supplementary Materials

Supplementary Materials Table S1: information for the expression vectors used in this study. Table S2: information for the primer in this study. Fig. S1: TEX10 promotes efficiency of NHEJ.
