# Supplementary material for: TEX10 Promotes the Tumorigenesis and Radiotherapy Resistance of Urinary Bladder Carcinoma by Stabilizing XRCC6
Source: J Immunol Res. 2021 Dec 20;2021:5975893. doi: 10.1155/2021/5975893 (PMC8712183; doi:10.1155/2021/5975893)

**Table S1. Information for the expression vectors used in this study**

| Plasmids | Sources | Vectors |
| --- | --- | --- |
| Myc-DDK-RNF216 | Origene (RC209430) | pCMV6-Entry |
| Flag-RNF216 | Subcloned | pCDH-CMV-MCS-EF1-Puro |
| Myc-DDK-DIAPH3 | Origene (RC221432) | pCMV6-Entry |
| Flag- DIAPH3 | Subcloned | pCDH-CMV-MCS-EF1-Puro |
| HA-RNF216 | Subcloned | pCDH-CMV-MCS-EF1-Puro |
| Ubiquitin | Hedgehogbio | pCMV |
| V5-Ubiqutin | Subcloned | pCMV6-Entry |

**Table S2. Information for the primer in this study**

| Plasmids | Primers | Sequences |
| --- | --- | --- |
| Flag-RNF216 | Forward | GGAATTCATGGAAGAGGGAAACAAC |
|  | Reverse | CAGCCGCGGCATCGCTTCGGATCCCG |
| Flag- DIAPH3 | Forward | GGAATTCATGGAACGGCACCAGCCG |
|  | Reverse | GCAAGATTACGAGCTTTAGGATCCCG |
| V5-Ubiquitin | Forward | CGGATCCATGCAGATCTTCGTGAAA |
|  | Reverse | CGCCTGAGGGGTGGCTGTGAATTCCG |

**Fig. S1.** TEX10 promotes efficiency of NHEJ

(A-B) Indicated cells were subjected to real-time PCR analysis. (C) J82 cells was treated with increasing doses of IR and subjected to survival assays by CCK8 assay. ***, p < 0.001.


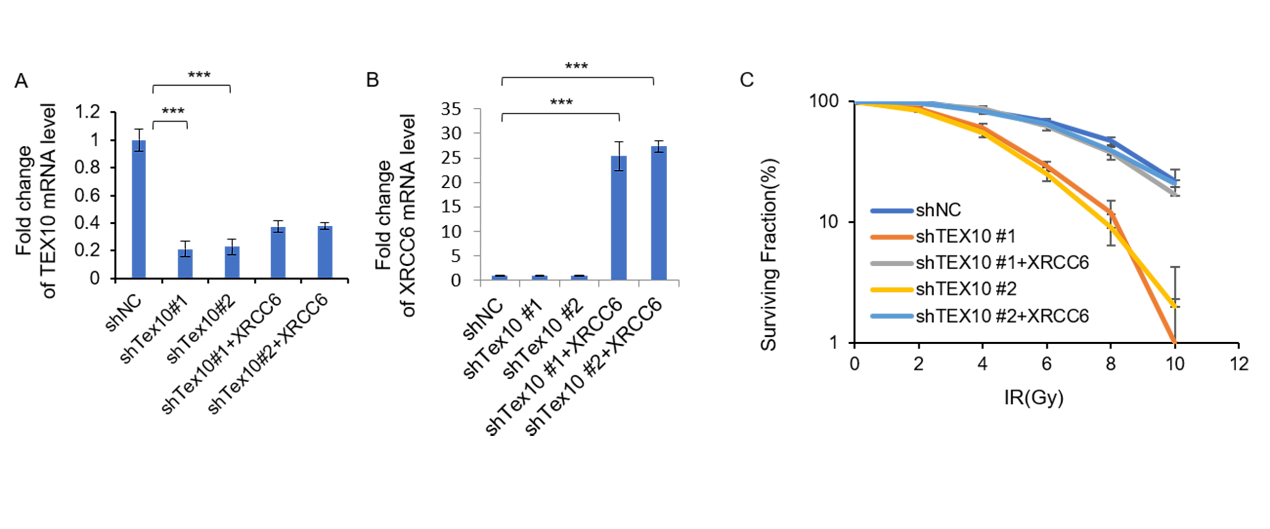

Supplement: Supplementary Materials — Table S1: information for the expression vectors used in this study. Table S2: information for the primer in this study. Fig. S1: TEX10 promotes efficiency of NHEJ. [file 5975893.f1.docx]
